# A Preliminary Study of the Cross-Reactivity of Canine MAGE-A with Hominid Monoclonal Antibody 6C1 in Canine Mammary Gland Tumors: An Attractive Target for Cancer Diagnostic, Prognostic and Immunotherapeutic Development in Dogs

**DOI:** 10.3390/vetsci7030109

**Published:** 2020-08-10

**Authors:** Wanwisa Srisawat, Boondarika Nambooppha, Kidsadagon Pringproa, Atigan Thongtharb, Worapat Prachasilchai, Nattawooti Sthitmatee

**Affiliations:** 1 Department of Veterinary Bioscience and Veterinary Public Health, Faculty of Veterinary Medicine, Chiang Mai University, Chiang Mai 50100, Thailand; wanwisasrisawat@gmail.com (W.S.); boondarika.n@gmail.com (B.N.); kidsadagon@hotmail.com (K.P.); 2 Veterinary Diagnostic Center, Faculty of Veterinary Medicine, Chiang Mai University, Chiang Mai 50100, Thailand; 3 Department of Companion Animal and Wildlife Clinic, Faculty of Veterinary Medicine, Chiang Mai University, Chiang Mai 50100, Thailand; attvet62@hotmail.com (A.T.); liverpoolpat@yahoo.com (W.P.); 4 Research Center in Bioresources for Agriculture, Industry and Medicine, Chiang Mai University, Chiang Mai 50200, Thailand; 5 Center of Excellence for Veterinary Bioscience, Chiang Mai University, Chiang Mai 50100, Thailand

**Keywords:** canine mammary gland tumors, human monoclonal MAGE-A antibody 6C1, immunohistochemistry, melanoma associated antigen-A, tumor marker

## Abstract

Melanoma-associated antigen-A (MAGE-A), a family of cancer/testis antigens, has been recognized as a potential target molecule for cancer immunotherapy. However, there has been very little information available with regard to this antigen in dogs. This study aimed to investigate the expression of MAGE-A in canine mammary gland tumors (CMTs) using immunohistochemistry and immunoblotting with human monoclonal MAGE-A antibody 6C1. The present study has provided evidence of cross-reactivity of the canine MAGE-A expression with the human MAGE-A antibody in CMTs. The MAGE-A antigens were expressed in moderate- and high-grade malignant CMTs (22.22%, 2/9), but no expression was observed in benign CMTs. The immunohistochemical staining of canine MAGE antigen in CMT cells showed nuclear and nuclear–cytoplasmic expression patterns that may be involved with the mitotic cell division of tumor cells. Molecular weights of the canine MAGE-A antigen presented in this study were approximately 42–62 kDa, which were close to those of other previous studies involving humans and dogs. The findings on this protein in CMTs could supply valuable oncological knowledge for the development of novel diagnostic, prognostic and immunotherapeutic tumor markers in veterinary medicine.

## 1. Introduction

Mammary tumors are common type of neoplasms observed in women and bitches [1,2]. In human medicine, the melanoma-associated antigen family A (MAGE-A)—which belongs to a specific family of cancer/testis antigens (CTA)—has been described as an attractive target for cancer immunotherapy [3,4]. MAGE antigen was first discovered in the human melanoma cell line [5]. Later, MAGE antigens were found to be present in other types of tumors. To date, MAGE antigens have been classified as MAGE Type 1 (MAGE-1) and MAGE Type 2 (MAGE-2) depending on the relevant characteristics of cell expression [6,7]. The MAGE-1 is a group of MAGE antigens that were not expressed in normal tissue cells during puberty, except for certain germ cells such as testicular cells or placental cells. In spite of this, the MAGE-2 is a group of MAGE antigens that are known to be present in normal tissues. The human *MAGE-1* gene family is composed of *MAGE-A, MAGE-B* and *MAGE-C* genes, which are distinguished by the location of the X Chromosome.

*MAGE-A* could promote cell progression by functioning as a transcriptional regulator, lead to tumor-forming. Therefore, the expression of this gene needs to be controlled by the regulation of the body system. The DNA methylation of CpG rich sites of DNA is one of the main mechanisms of *MAGE-A* repression in other tissue cells except for germ cells. This paradigm is supported by several studies of the upregulation of *MAGE-A* mRNA expression of culture cells results when these cells were induced with the promoter demethylation inhibitor reagent (5-aza-2-deoxycytidine) lead to S phase of the cell cycle was extended. In contrast, the histone deacetylation, a mechanism of post-translation modification, was suggested that it plays a role in *MAGE-A* gene stimulation of the hypermethylation cells [8].

Moreover, the unique MAGE-A intracellular epitopes can be presented on the cell surface by major histocompatibility complex Class 1 (MHC Class 1) molecules by processing of the proteasome, which can function as specific epitopes for target therapies [4,9]. These epitopes could be recognized by cytotoxic T lymphocyte (CTL) and can contribute to the stimulation of the immune system [10,11,12,13]. MAGE-A expression in cancer cases has been reported in brain tumors, lung tumors, and in cases pancreatic cancer, ovarian cancer and breast cancer [14,15,16]. In human breast cancer, the expression of this antigen has been related to incidences of poor prognosis outcomes [17,18].

In the context of veterinary medicine, canine mammary tumors (CMT) have been diagnosed in more than 50% of all tumor cases in intact female dogs [19,20]. These tumors have been observed to display clinical and molecular similarities to spontaneous tumors found in humans with regard to hormonal etiology, the age of onset and in terms of the course of the disease [21,22]. The rate of recurrence has been recorded at up to 58% after surgery, contributing to poor prognoses and increased incidences of death in cases of high-grade malignant CMTs [23,24,25]. Additionally, chemotherapy has yielded limited success in terms of tumor control and has been associated with numerous side effects [26,27,28,29]. These findings emphasize the requirements of new choices for CMT treatments. Tumor-infiltrating immune cells are commonly observed in CMT tissues, and they appear to play a significant role in the tumor microenvironment by interacting with tumor cells that contribute to the disease progression and clinical outcome. For example, increased CD4+/CD8+ T cell ratios were associated with decreased survival in dogs with malignant CMTs, and the number of Foxp3+ regulatory T cells was increased in aggressive CMTs [30,31]. These studies suggest that the immunosuppressive components provide clinical implications for CMTs. The promising strategy of immunotherapy involves the stimulation of the function of the lymphocytes, mainly the cytotoxic T lymphocytes and the natural killer cells, act in response to and in eradication of the cancer cells [32].

Previous studies have identified the pathologic characteristics of CMTs as being similar to human breast cancers [33,34]. However, the accumulation of data on MAGE antigens expression in CMT has been limited. Therefore, this study aimed to explore the expression of MAGE as the target antigen for further diagnostic, prognostic and immunotherapeutic development in CMTs using immunohistochemistry assay (IHC). This assay has been established by the principles of the specific antibodies that are attached to the target antigens of the tissues [35]. Furthermore, this study focused on allocating MAGE expression and the relationship of the expression, while the histological grade of the CMT we have referred to has become a clinical prognosis index.

## 2. Materials and Methods

### 2.1. Tissue Samples

Formalin-fixed paraffin-embedded tissues (FFPE) of CMT tissues were obtained from the Veterinary Diagnostic Center, Chiang Mai University. FFPE tissues were cut into 3–4 µm segments, placed on glass slides and then stained with hematoxylin and Eosin solutions (Richard-Allan Scientific, San Diego, CA, USA) according to the manufacturer’s instructions. The canine mammary tumor were classified according to the World Health Organization classification of canine mammary tumor 1999 [36]. Latter, the CMT tissues of carcinomas were scored of tubule formation, nuclear criteria and mitotic figures according to the histological malignancy grade classification of 2002; well-differentiated, Grade 1, intermediately to moderately differentiated, Grade 2 and poorly differentiated, Grade 3 [37].

Normal testicular tissue samples were obtained from Chiang Mai University Small Animal Hospital. The fresh tissue was separated into two groups. In the first group, tissue samples were cut into small pieces of approximately 0.5–1 cm in diameter and fixed in 10% neutral buffered formalin solution for 16 h. The tissue samples were then transferred into the cassettes and this step was followed by a processing cycle using a tissue processor machine for paraffin impregnation according to the manufacturer’s instructions (Leica Bio system, Wetzlar, Germany). The processed tissues were then embedded in paraffin blocks and kept at room temperature. The other group of testicular tissue samples used for the protein extraction was pour with the liquid nitrogen and kept in −70 °C until being used.

### 2.2. Immunohistochemistry

The method applied in this study was adapted from that which has been described previously [38]. FFPE tissues were cut into sections of 3–4 µm in thickness. The tissue slides were rehydrated 2 times by immersion in xylene for 5 min each, 2 times in absolute alcohol for 2 min each, 2 times in 90% alcohol for 2 min each, 2 times in 80% alcohol for min each, 2 times in 70% alcohol for 2 min each and once in tap water for 5 min, respectively. Pretreatment was conducted with heat-induced epitope retrieval (HIER) of citric acid buffer at a pH value of 6 at 800 watts 3 times for 10 min each and then washed with distilled water for 30 s. To eradicate the natural peroxidase enzyme, each prepared tissue sample had 3% hydrogen peroxide solution (H_2_O_2_) dropped on it at room temperature for 10 min, and they were then washed 3 times with phosphate buffer solution (PBS) for 5 min each. A nonspecific reaction was then blocked on each tissue using 5% of bovine serum albumin (BSA) in PBS at room temperature for 1 h. Subsequently, the samples were washed 3 times with phosphate buffer solution with 0.1% Tween-20 (PBST) for 5 min each.

The mouse anti-human monoclonal antibody 6C1 (Cat No. 35-6300, Lot No. TG273242, Invitrogen, Carlsbad, CA, USA) which previous descript that it made of the conserved domain of human MAGE-A protein, was selected to apply in this study according to a high percentage of protein identity matching between canine and human MAGE (Table 1). The primary antibody dilution values of 1:100, 1:200, 1:400, 1:800 and 1:1000 in PBS were tested for the appropriate concentrations of the antibody. The tissue samples were incubated with 6C1 antibody at 4 °C overnight in the humidity chamber and then washed 3 times with PBST for 5 min each. Additionally, biotinylated anti-mouse secondary antibody (Thermo Fisher Scientific, Waltham, MA, USA) at a dilution of 1:200 in PBS was added to each slide, and the specimens were incubated at room temperature for 2 h and then washed 3 times with PBST for 5 min each. Furthermore, avidin-biotin-complex system reagent (Thermo Scientific) was added to each slide, and then the specimens were incubated at room temperature for 50 min and washed 3 times with PBST for 5 min each. The visualized detection process was administered to the tissue samples with 0.02% DAB and 0.02% H_2_O_2_ in PBS for 5–10 min, and the tissue samples were then washed with tap water in order to stop the reaction. The nuclei of the cells were stained with hematoxylin solution for 5 min. They were then rinsed with tap water and rehydrated with 70% alcohol for 2 min, 80% alcohol for 2 min, 90% alcohol for 2 min and absolute alcohol for 2 min, respectively. The slides were then immersed in xylene for 5 min. The solvent-based medium (Thermo Scientific) was then dropped onto them and each slide was covered with a coverslip. Brown colored staining areas were detected under a light microscope. The testicular sample was used as a positive control in detecting the MAGE-A antigen. The 6C1 monoclonal antibody was replaced by the normal mouse serum to serve as the negative control.

### 2.3. Protein Extraction

Each 0.2 g sample of the frozen testicular tissue or mammary gland tumor tissue was poured with the liquid nitrogen. The frozen specimen was ground until being the powder-like particles and mixed with 700 mL of Tris-HCl (pH = 9) lysis buffer and 1 µL of cocktail protease inhibitor. The samples were then incubated on ice for 2 h. After that, each tissue sample was centrifuged at 14,000× *g* in 4 °C for 20 min. Consequently, the middle phase layer of lysate was transferred to a new 1.5 mL microbial free microcentrifuge tube. The protein concentrations were then measured by BCA Protein Assay and kept in −20 °C until being used.

Protein extraction of the FFPE of CMT tissue sections was performed following the method described by Guo et al. [40]. Briefly, the FFPE of CMT tissue sections were placed in 1.5-mL microcentrifuge tubes and deparaffinized by xylene (Fisher Scientific, Pittsburgh, PA, USA) for 10 min at room temperature. Each tissue sample was centrifuged at 14,000× *g* for 3 min and then the incubation/centrifugation steps were repeated two times. The deparaffinized tissue pellets were then rehydrated with ethanol and resuspended using Tris-HCl (pH = 9) lysis buffer with 2% (*w/v*) SDS. The samples were incubated on ice for 5 min. They were then vortexed and heated at 100 °C for 20 min. Afterwards, the samples were incubated at 80 °C for 2 h. The protein concentrations were then measured by BCA Protein Assay (Pierce Chemicals Co., Rockford, IL, USA).

### 2.4. SDS-PAGE and Western Immunoblotting Analysis

Each 15 µg of total proteins were mixed with 2× Lemmli buffers at a ratio of 1:1 and boiled at 95 °C for 7 min. The proteins were separated by 12.5% sodium dodecyl sulfate–polyacrylamide gel electrophoresis (SDS-PAGE) at a voltage of 100 for 1.5 h. The proteins separated from the polyacrylamide gel were transferred onto the nitrocellulose membrane by electroblotting technique. The nonproteins embedded in the membrane area were then coated with the blocking buffer (5% BSA in PBS) for 1 h. The membrane was washed 3 times with PBST for 5 min each. The mouse anti-human monoclonal antibody 6C1 was diluted in the blocking buffer (1:1000) and poured onto the membrane, which was then incubated at 4 °C overnight. The membrane was then washed 3 times for 5 min each with PBST. The HRP-conjugated goat anti-mouse secondary antibody was diluted in PBS (1:2500), poured onto the membrane and then incubated at room temperature for 2 h. The membrane was washed again 3 times for 5 min each with PBST. The visualized detection assay was performed by transferring the membrane into 0.02% DAB and 0.02% H_2_O_2_ in PBS for 5–10 min. The membrane was then washed with tap water in order to stop the reaction. A brown color band was displayed to indicate a positive band.

### 2.5. Data Analysis

Type and scoring of CMTs were determined and characterized by a specialist and two well-trained observers. The immunohistochemistry (IHC) image analysis toolbox of the ImageJ analysis software was used to determine the staining results. No staining or weak staining in <5% of cells was defined as 0, weak staining in at least 5% as 1, moderate staining in up to 50% as 2 and moderate staining in >50% of cells and strong staining of any percentage of the cells as 3 [41]. Grade 0 was defined as negative and Grade 1–3 were defined as positive. The results of expression were determined using descriptive analysis.

## 3. Results

### 3.1. CMT Classification and Grading

From a total of 11 CMT samples, there were 2 benign CMT samples and 9 malignant CMT samples included in this study. All benign CMTs were classified in Grade 1 by TNM score. However, almost all malignant CMTs were classified in Grade 3 (88.9%, 8/9) by TNM with the exception of a fibrosarcoma tumor that was obtained from a 10-year-old mixed-breed dog listed in Table 2. This tumor was classified as Grade 2 by TNM score. The histological grades of the carcinomas were classified as follows; 16.70% (1/6) of Grade 1, 33.30% (2/6) of Grade 2 and 50% (3/6) of Grade 3. All CMT samples in this study were collected from 5- to 14-year-old female dogs of small and medium-sized breeds (Table 2 and Table 3).

### 3.2. Canine MAGE-A Antigens Recognized by Human MAb 6C1

#### 3.2.1. MAGE-A Antigens Recognized by MAb 6C1 in Canine Testicular Tissue

**Figure 1:**
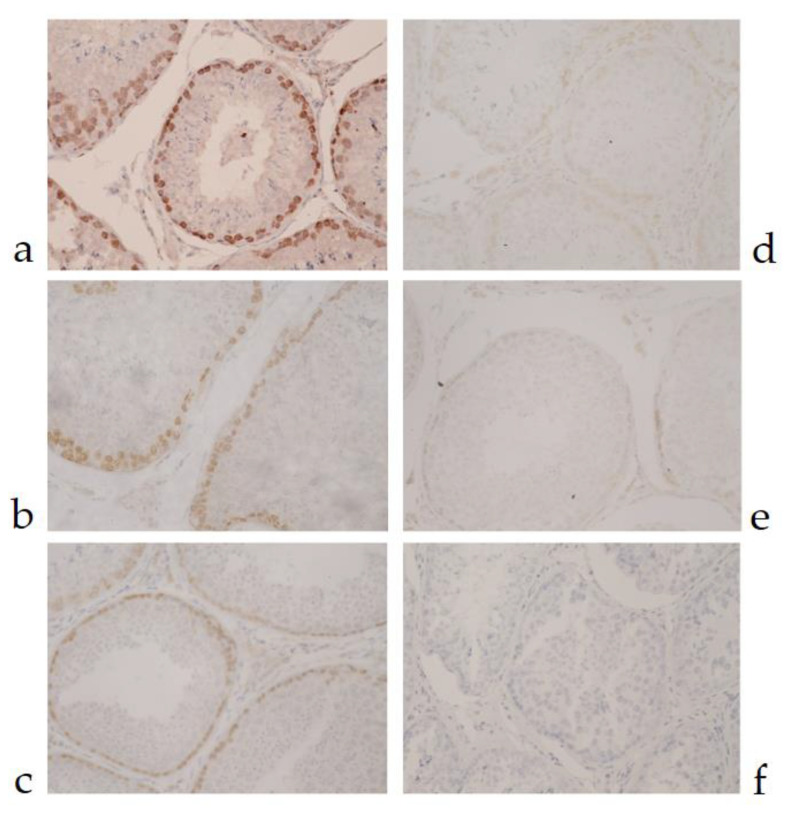
Positive cross-reactivity of MAb 6C1, testis and canines. The detection of MAGE antigen in canine testicular tissue sections at different dilutions using IHC method (40×). (**a**–**e**) MAb 6C1 in phosphate buffer solution (PBS) in dilutions of 1:100, 1:200, 1:400, 1:800 and 1:1000, respectively; (**f**) negative control (normal mouse serum in PBS in a dilution of 1:200).

**Figure 2:**
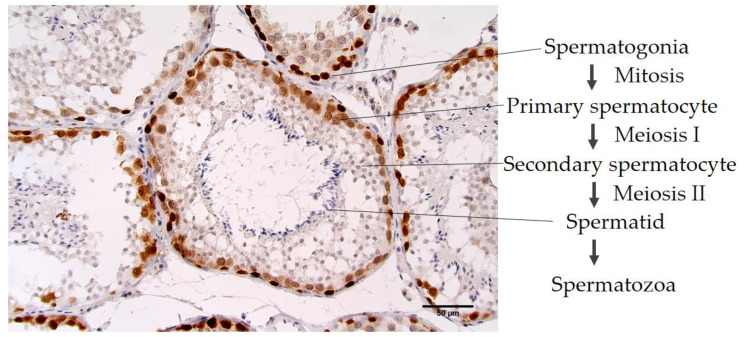
Positive cross-reactivity of MAb 6C1, testis and canines. The location of MAGE expression in canine testicular tissue sections (40×).

MAb 6C1 in a dilution of 1:100 to 1:1000 in PBS was able to detect MAGE antigens in canine testicular tissue (Figure 1). In normal testicular tissue, MAGE antigens were detected strongly and specifically within the seminiferous tubules (Figure 2). Spermatogonia were the most intensely labeled cells. Primary spermatocytes were found to be immunoreactive, but weaker staining was consistently revealed when compared with the spermatogonia. Spermatids, Sertoli cells and spermatozoa reacted negatively with human MAGE-A 6C1 MAb. This antigen is mainly considered a nuclear antigen.

#### 3.2.2. MAGE-A Antigens Recognized by MAb 6C1 in CMT Tissues

**Figure 3:**
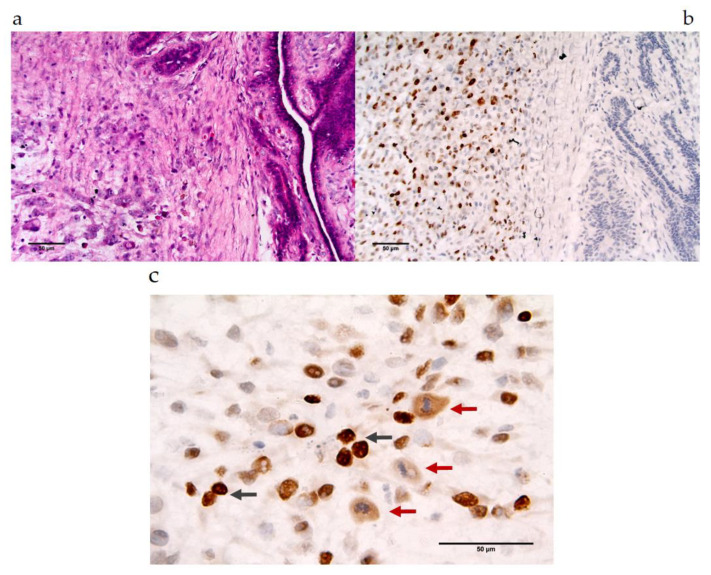
Positive cross-reactivity of MAb 6C1, mammary gland tumors and canines. Location of MAGE expression in the complex carcinoma type of malignant mammary gland tumor section Ma1: (**a**) HE stained, 40×; (**b**) IHC-stained-positive, 40×; (**c**) IHC-stained-positive, 100× black arrows are representative of nuclear expression pattern; red arrows are representative of nuclear–cytoplasmic expression pattern.

**Figure 4:**
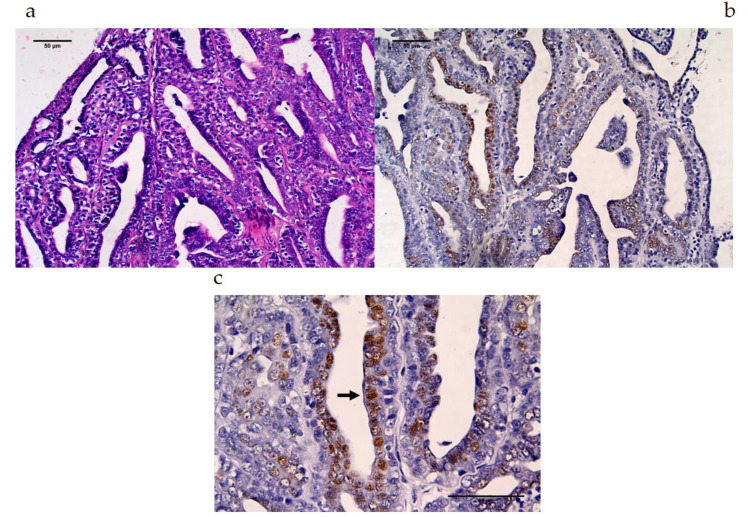
Positive cross-reactivity of MAb 6C1, mammary gland tumors and canines. Location of MAGE expression in the simple tubular carcinoma type of malignant mammary gland tumor section Ma2: (**a**) HE stained, 40×; (**b**) IHC-stained-positive, 40×, (**c**) IHC-stained-positive, 100×, black arrows are representative of nuclear expression pattern.

The 6C1 MAb antibody at a dilution of 1:100 was applied to CMT sections. The immunolabeling for MAGE-A is presented in Table 2 and Table 3. There was no MAGE-A expression in the benign CMT tissues that was detected by human Mab 6C1, while 22.22% (2/9) were observed in the malignant CMT tissues. The MAGE-A antigens were primarily detected in the nucleus (Figure 3 and Figure 4) of the malignant CMT cells. Moreover, some CMT cells at the mitosis stage that revealed no nuclear membrane were stained for MAGE-A in both the nucleus and cytoplasm (Figure 3c).

### 3.3. Molecular Mass and Immunoreactivity of Canine MAGE-A Antigen

**Figure 5:**
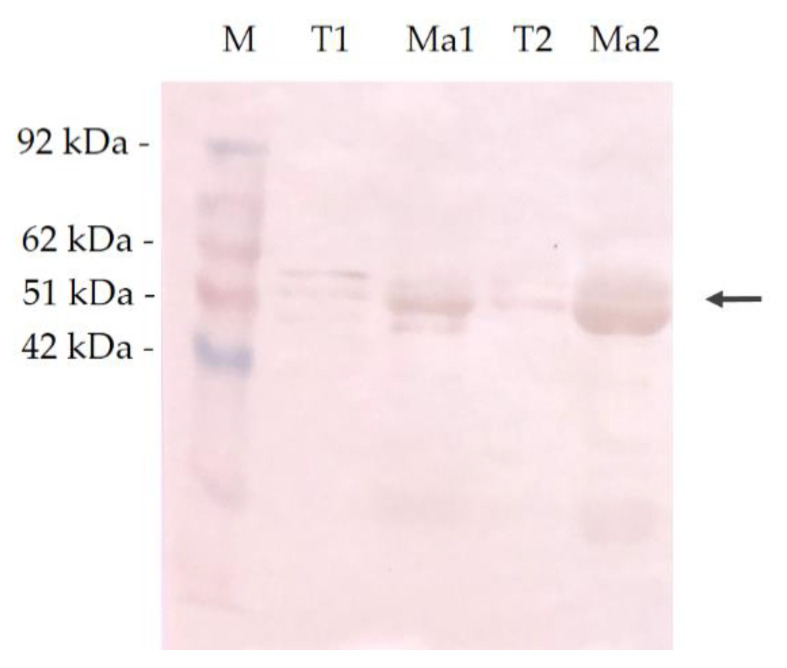
Positive cross-reactivity of MAb 6C1, testis, mammary gland tumors and canines. Western blot analysis of testicular and CMT cells with human anti-MAGE Mab 6C1. Black arrows indicate the positive cross-reactivity at approximately 42–62 kDa; M—protein marker (kDa); T1; T2—protein of testicular samples; Ma1; Ma2—protein of IHC positive malignant CMT sample.

The binding capacity of human MAb 6C1 to MAGE-A antigen expressed in testicular tissues and positive immunolabeling CMT tissues was evaluated using western blotting analysis. The reactivity was observed at approximately 42–62 kDa (Figure 5).

## 4. Discussion

MAGE-A is an antigen recognized by cytotoxic T lymphocyte (CTL) via the presentation of the MHC Class 1 and contributes to stimulating the immune system and eradicating specific MAGE-A expressed cancer cells [4,5,11,12,13]. In spite of the fact that MAGE-A belongs to CTA, a lack of MHC Class 1 in germ cells can lead to MAGE-A being unrecognizable to CTLs and may have no influence on autoimmunity [42]. This would imply that this antigen could be an ideal target for cancer immunotherapy [3]. Although studies of MAGE-A in human cancers have been conducted, information regarding this antigen in promoting canine cancer is very limited. Likewise, studies on targeted cancer therapy for canine cancers, which are similar types to human cancers, are lacking [43]. The present study revealed the relative ability of the human 6C1 monoclonal Antibody (MAb), which has been shown to recognize human MAGE-A1, -A2, -A3, -A4, -A6, -A10 and -A12, to be applied on CMTs [41]. Furthermore, the expression of canine MAGE-A in CMT samples showed the possibility of it being an attractive target for diagnostic, prognostic and immunotherapeutic development in veterinary medicine.

The immunohistochemical results presented in Figure 2 show the expression of canine MAGE-A antigens in normal testicular tissue; the nucleus (nuclear expression pattern) in the spermatogonia and both the nucleus and the cytoplasm (nuclear–cytoplasmic expression pattern) in the primary spermatocytes. Contrastingly, the secondary spermatocytes, spermatids and the spermatozoa were found to produce negative results of MAGE-A. The nuclear expression pattern in this study was similar to the pattern observed in previous studies involving MAGE antigens with recognized MAb 6C1 in human testicular tissue [44]. Additionally, the other sequences of MAGE, such as MAGE-A MAb 57B (anti-MAGE-A3, -A4, -A6 and -A12) and MAGE-4 MAb R5 (anti- MAGE-4), revealed an identical immunoreactivity expression pattern as was found in this study [17,18]. In contrast, previous studies revealed that the canine MAGE antigen could be displayed in the nucleus and cytoplasm in all stages of spermatogenesis including in the spermatid cells [38]. According to our results, canine MAGE-A may plays an essential function on the spermatogenesis mainly on the phase of spermatogonia to primary spermatocyte. One of the explanations of the nuclear pattern related to the functional of MAGE-A detection in spermatogonia is related to the transcriptional regulator protein. P53, a cell division checkpoint, is located in the nucleus of cells and bind directly with the DNA [45]. This protein is absent in spermatogonia relate to mitotic division continue progression [46]. The role of MAGE-A was suggested that it may inhibit p53 function by interacting with three distinct peptides each of which is located within the DNA binding surface of the core domain of p53 and encompasses amino acids that are critical for site-specific DNA binding [47]. However, the pattern and location of the expression of antigens on the cells may vary depending on the function of the antigen and the sub-type of the canine antigen that could present at that moment in time. Importantly, the issue of MAGE antigen expression pattern in dogs requires further investigation.

Previous studies revealed MAGE-A in canine melanomas including oral, cutaneous, eyelid and interdigital melanomas [37]. This study revealed that 22% (2/9) of malignant CMT samples, which were classified in moderately grade and poorly grade carcinoma groups, displayed MAGE-A expression. The molecular weights of the canine MAGE-A antigen of CMT tissues expressed in this study were approximately 51–62 kDa and were found to be close to those in studies involving humans and in one recent study involving dogs. These molecular weights have been described as 45–50 kDa [38,39,42,43]. The expression level in the CMTs was similar to that of another recent study on the MAGE-A expression with MAb 6C1 in human breast cancer cases, which was 17% and has been associated high-grade malignancy [16]. The immunohistochemical staining of canine MAGE antigen in CMT samples was strongly correlated with a nuclear expression pattern and was detected in some tumor cells of the tissue section (Figure 3 and Figure 4). This pattern of expression in tumor cells was similar to that of previous studies on human breast cancer and lung cancer, in which the expression was dominant in the nucleus [16,17]. Interestingly, the tumor cells that were expected to be in the mitotic process revealed moderate expression levels of the nuclear–cytoplasmic expression pattern. MAGE-1 has been compromised in the regulation of p53 and E2F1 transcription factors and can promote tumor cell proliferation [46,48,49,50]. The function of which occurs at the interphase stage as a checkpoint of cell division. The results support the possibility that canine MAGE-A antigen expression, as was observed in the present study, could play a role in the interphase stage of mitotic cell division of CMT cells.

The tissue section slides of CMT that were composed of the various types and stages of the cancerous cells were not constant in terms of the number of divided cells. The malignant CMT cells had a high level of divided cells when compared with the benign CMTs. This may be the reason for the positive results that occurred in the moderate- and high-grade specimens of malignant CMTs; however, no expression was revealed in the benign CMTs. The data suggest that the expression levels of canine MAGE-A antigens in cancer cells were related to the proliferation of the tumor cells that are involved with mitotic cell division. The findings of this protein in CMTs are considered an important step in targeted-cancer therapeutic development in the veterinary field. For further study, the MAGE-A subtype expression in CMTs associated with a few other markers related to tumor aggressiveness or apoptosis, such as Caspase 3, PCNA or ki-67 and the function of this protein family should be continue studied in a large number of samples to fully clarify whether MAGE-A is involved in apoptosis or cell proliferation during tumor growth. Additionally, the study of MAGE-A are needed involving other neoplasms in dogs for the development of optimum diagnosis, prognosis and effective treatment.

## 5. Conclusions

Our study has provided evidence of the canine MAGE-A expression that is associated with cross-reactivity in human MAGE-A antibodies in CMTs. The results indicate that the expression pattern in CMT cells was restricted to the nuclear and nuclear–cytoplasmic patterns that may be involved with the mitotic cell division. These findings support the suggestion that MAGE-A is an attractive antigen and could supply valuable oncological knowledge for the development of novel immunotherapeutic tumor markers in the veterinary field.

## Figures and Tables

**Table 1:** Protein sequence and percent homology of 6C1 immunogen with human MAGE-A10 and canine MAGE-A8,-A9 and -A10 of National Center for Biotechnology Informatio (NCBI) databases [39].

Protein Name	NCBI No.	Peptide Sequences											Percent Homology
6C1immunogen	–	S	D	P	A	R	Y	E	F	L	W	G	–
hMAGE-A10	NP_001238757.2	S	D	P	A	R	Y	E	F	L	W	G	100
cMAGE-A8	XP_005642019.3	S	D	P	A	R	Y	E	F	L	W	G	100
cMAGE-A9	XP_005642020.1	S	N	P	A	R	Y	E	F	L	W	G	90.1
cMAGE-A10	XP_022272024.1	S	D	P	A	C	Y	E	F	L	W	G	90.1

**Table 2:** Results of canine MAGE antigen recognized human MAb 6C1 in benign CMT tissues.

No.	Age (Years)	Breed	Type of Tumor	TNM Score	MAGE Expression Score
				(1–4)	(0–3)
B1	5	Pomeranian	Benign mix tumor	1	0
B2	10	Mixed	Complex adenoma	1	0

**Table 3:** Results of canine MAGE antigen recognized human MAb 6C1 in malignant CMT tissues.

No.	Age (Years)	Breed	Type of Tumor	TNM Score	Histological Grade	MAGE Expression Score
				(1–4)	(1–3)	(0–3)
Ma1	10	Chihuahua	Complex carcinoma	3	3	2
Ma2	10	Mixed	Simple tubular carcinoma	3	2	2
Ma3	10	Mixed	Anaplastic carcinoma	3	3	0
Ma4	12	Pomeranian	Complex carcinoma	3	1	0
Ma5	12	Poodle	Fibroadenocarcinoma	3	–	0
Ma6	14	Miniature	Carcinosarcoma	3	–	0
Ma7	12	Mixed	Simple Solid carcinoma	3	2	0
Ma8	14	Cocker Spaniel	Simple Solid carcinoma	3	3	0
Ma9	10	Mixed	Fibrosarcoma	2	–	0

## References

[1] Salas Y., Márquez A., Diaz D., Romero L. (2015). Epidemiological study of mammary tumors in female dogs diagnosed during the period 2002–2012: A growing animal health problem. PLoS ONE.

[2] Abdelmegeed S.M., Mohammed S. (2018). Canine mammary tumors as a model for human disease. Oncol. Lett..

[3] Sang M., Lian Y., Zhou X., Shan B. (2011). MAGE-A family: Attractive targets for cancer immunotherapy. Vaccine.

[4] Schooten E., Di Maggio A.D., Van Bergen En Henegouwen P.M.P., Kijanka M.M. (2018). MAGE-A antigens as targets for cancer immunotherapy. Cancer Treat. Rev..

[5] Van der Bruggen P., Traversari C., Chomez P., Lurquin C., De Plaen E., Van den Eynde B., Knuth A., Boon T. (1991). A gene encoding an antigen recognized by cytolytic T lymphocytes on a human melanoma. Science.

[6] Barker P.A., Salehi A. (2002). The MAGE proteins: Emerging roles in cell cycle progression, apoptosis, and neurogenetic disease. J. Neurosci. Res..

[7] Caballero O.L., Chen Y.T. (2009). Cancer/testis (CT) antigens: Potential targets for immunotherapy. Cancer Sci..

[8] Beumer T.L., Roepers-Gajadien H.L., Gademan I.S., Van Buul P.P.W., Gil-Gomez G., Rutgers D.H., De Rooij D.G. (1998). The role of the tumor suppressor p53 in spermatogenesis. Cell Death Differ..

[9] Vamolri D., Gildaei U., Servis C., Dunbar P.R., Cerottini J., Romeo P., Cerudoro V., Lévy F. (1999). Modulation of proteasomal activity required for the generation of a cytotoxic T lymphocyte-defined peptide Derived from the Tumor Antigen MAGE-3. J. Exp. Med..

[10] Duffour M.T., Chaux P., Lurquin C., Cornelis G., Boon T., Van der Bruggen P. (1999). A MAGE-A4 peptide presented by HLA-A2 is recognized by cytolytic T lymphocytes. Eur. J. Immunol..

[11] Pascolo S., Schirle M., Gückel B., Dumrese T., Stumm S., Kayser S., Moris A., Wallwiener D., Rammensee H.G., Stevanovic S. (2001). A MAGE-A1 HLA-A A*0201 epitope identified by mass spectrometry. Cancer Res..

[12] Ottaviani S., Colau D., Van der Bruggen P., Van der Bruggen P. (2006). A new MAGE-4 antigenic peptide recognized by cytolytic T lymphocytes on HLA-A24 carcinoma cells. Cancer Immunol. Immun..

[13] Chinnasamy N., Wargo J.A., Yu Z., Rao M., Frankel T.L., Riley J.P., Hong J.J., Parkhurst M.R., Feldman S.A., Schrump D.S. (2011). A TCR targeting the HLA-A*0201-restricted epitope of MAGE-A3 recognizes multiple epitopes of the MAGE-A antigen superfamily in several types of cancer. J. Immunol..

[14] Kuramoto T. (1997). Detection of MAGE-1 tumor antigen in brain tumor. Kurume Med. J..

[15] Sugita M., Geraci M., Gao B., Powell R.L., Hirsch F.R., Johnson G., Lapadat R., Gabrielson E., Bremnes R., Bunn P.A. (2002). Combined use of oligonucleotide and tissue microarrays identifies cancer/testis antigens as biomarkers in lung carcinoma. Cancer Res..

[16] Chen Y.T., Ross D.S., Chiu R., Zhou X.K., Chen Y.Y., Lee P., Hoda S.A., Simpson A.J., Old L.J., Caballero O. (2011). Multiple cancer/testis antigens are preferentially expressed in hormone-receptor negative and high-grade breast cancers. PLoS ONE.

[17] Curigliano G., Viale G., Ghioni M., Jungbluth A.A., Bagnardi V., Spagnoli G.C., Neville A.M., Nolè F., Rotmensz N., Goldhirsch A. (2011). Cancer-testis antigen expression in triple-negative breast cancer. Ann. Oncol..

[18] Ayyoub M., Scarlata C.M., Hamaï A., Pignon P., Valmori D. (2014). Expression of MAGE-A3/6 in primary breast cancer is associated with hormone receptor negative status, high histologic grade, and poor survival. J. Immunother. Cancer.

[19] Moe L. (2001). Population-based incidence of mammary tumours in some dog breeds. J. Reprod. Fertil. Suppl..

[20] Merlo D.F., Rossi L., Pellegrino C., Ceppi M., Cardellino U., Capurro C., Ratto A., Sambucco P.L., Sestito V., Tanara G. (2008). Cancer incidence in pet dogs: Findings of the animal tumor registry of Genoa, Italy. J. Vet. Intern. Med..

[21] Bronson R.T. (1982). Variation in age at death of dogs of different sexes and breeds. Am. J. Vet. Res..

[22] Dobson J.M., Samuel S., Milstein H., Rogers K., Wood J.L. (2002). Canine neoplasia in the UK: Estimates of incidence rates from a population of insured dogs. J. Small Ani. Pract..

[23] Yamagami T., Kobayashi T., Takahashi K., Sugiyama M. (1996). Influence of ovariectomy at the time of mastectomy on the prognosis for canine malignant mammary tumours. J. Small Ani. Pract..

[24] Karayannopoulou M., Kaldrymidou E., Constantinidis T.C., Dessiris A. (2005). Histological grading and prognosis in dogs with mammary carcinomas: Application of a human grading method. J. Comp. Pathol..

[25] Stratmann N., Failing K., Richter A., Wehrend A. (2008). Mammary tumor recurrence in bitches after regional mastectomy. Vet. Sur..

[26] Karayannopoulou M., Kaldrymidou E., Constantinidis T.C., Dessiris A. (2001). Adjuvant post-operative chemotherapy in bitches with mammary cancer. J. Vet. Med. A Physiol. Pathol. Clin. Med..

[27] Simon D., Schoenrock D., Baumgärtner W., Nolte I. (2006). Postoperative adjuvant treatment of invasive malignant mammary gland tumors in dogs with doxorubicin and docetaxel. J. Vet. Int. Med..

[28] Marconato L., Lorenzo R.M., Abramo F., Ratto A., Zini E. (2008). Adjuvant gemcitabine after surgical removal of aggressive malignant mammary tumours in dogs. Vet. Comp. Oncol..

[29] Lavalle G.E., De Campos C.B., Bertagnolli A.C., Cassali G.D. (2012). Canine malignant mammary gland neoplasms with advanced clinical staging treated with carboplatin and cyclooxygenase inhibitors. In Vivo.

[30] Estrela-Lima A., Araújo M.S.S., Costa-Neto J.M., Teixeira-Carvalho C., Barrouin-Melo M., Cardoso S.V., Martins-Filho O.A., Serakides R., Cassali G.D. (2010). Immunophenotypic features of tumor infiltrating lymphocytes from mammary carcinomas in female dogs associated with prognostic factors and survival rates. BMC Cancer.

[31] Kim J.H., Chon S.K., Im K.S., Kim N.H., Cho K.W., Sur J.H. (2012). Correlation of Foxp3 positive regulatory T cells with prognostic factors in canine mammary carcinomas. Vet. J..

[32] Martínez-Lostao L., Anel A., Pardo J. (2015). How do cytotoxic lymphocytes kill cancer cells?. Clin. Cancer Res..

[33] Schneider R. (1970). Comparison of age, sex, and incidence rates in human and canine breast cancer. Cancer.

[34] Mottolese M., Morelli L., Agrimi U., Benevolo M., Sciarretta F., Antonucci G., Natali P.G. (1994). Spontaneous canine mammary tumors. A model for monoclonal antibody diagnosis and treatment of human breast cancer. Lab. Investig..

[35] Sternberger L.A., Hardy P.H., Cuculis J.J., Meyer H.G. (1970). The unlabeled antibody enzyme method of immunohistochemistry: Preparation and properties of soluble antigen-antibody complex (horseradish peroxidase-antihorseradish peroxidase) and its use in identification of spirochetes. J. Histochem. Cytochem..

[36] Misdorp W., Else R.W., Hellmén E., Lipscomb T.P. (1999). Histological Classification of Mammary Tumors of the Dogs and the Cats.

[37] Misdorp W., Meuten D.J. (2002). Tumor of the mammary gland. Tumors in Domestic Animals.

[38] Chen Y.C., Hsu W.L., Chiu C.Y., Liao J.W., Chang C.C., Chang S.C. (2013). Expression of MAGE--A restricted to testis and ovary or to various cancers in dogs. Vet. Immunol. Immunopathol..

[39] Rimoldi D., Salvi S., Schultz-Thater E., Spagnoli G.C., Cerottini J.C. (2000). Anti-MAGE-3 antibody 57B and anti-MAGE-1 antibody 6C1 can be used to study different proteins of the MAGE-A family. Int. J. Cancer.

[40] Guo H., Liu W., Ju Z., Tamboli P., Jonasch E., Mills G.B., Lu Y., Hennessy B.T., Tsavachidou D. (2012). An efficient procedure for protein extraction from formalin-fixed, paraffin-embedded tissues for reverse phase protein arrays. Proteome Sci..

[41] Van Diest P.J., Van Dam P., Henzen-Logmans S.C., Berns E., Van der Burg M.E., Green J., Vergote I. (1997). A scoring system for immunohistochemical staining: Consensus report of the task force for basic research of the EORTC-GCCG. European organization for research and treatment of cancer-gynaecological cancer cooperative group. J Clin Pathol..

[42] Fiszer D., Kurpisz M. (1998). Major histocompatibility complex expression on human, male germ cells: A review. Am. J. Reprod. Immunol..

[43] Addissie S., Klingemann H. (2018). Cellular Immunotherapy of canine cancer. Vet. Sci..

[44] Takahashi K., Shichijo S., Noguchi M., Hirohata M., Itoh K. (1995). Identification of MAGE-1 and MAGE-4 proteins in spermatogonia and primary spermatocytes of testis. Cancer Res..

[45] Aubrey B.J., Kelly G.L., Janic A., Herold M.J., Strasser A. (2018). How does p53 induce apoptosis and how does this relate to p53-mediated tumour suppression?. Cell Death Differ..

[46] Marcar L., MacLaine N.J., Hupp T.R., Meek D.W. (2010). Mage-A cancer/testis antigens inhibit p53 function by blocking its interaction with chromatin. Cancer Res..

[47] Lian Y., Meng L., Ding P., Sang M. (2018). Epigenetic regulation of MAGE family in human cancer progression-DNA methylation, histone modification, and non-coding RNAs. Clin. Epigenetics.

[48] Monte M., Simonatto M., Peche L.Y., Bublik D.R., Gobessi S., Pierotti M.A., Rodolfo M., Schneider C. (2006). MAGE-A tumor antigens target p53 transactivation function through histone deacetylase recruitment and confer resistance to chemotherapeutic agents. Proc. Natl. Acad. Sci. USA.

[49] Marcar L., Ihrig B., Hourihan J., Bray S.E., Quinlan P.R., Jordan L.B., Thompson A.M., Hup T.R., Meek D.W. (2015). MAGE-A cancer/testis antigens inhibit MDM2 ubiquitylation function and promote increased levels of MDM4. PLoS ONE.

[50] Su S., Minges J.T., Grossman G., Blackwelder A.J., Mohler J.L., Wilson E.M. (2013). Proto-oncogene activity of melanoma antigen-A11 (MAGE-A11) regulates retinoblastoma-related p107 and E2F1 proteins. J. Biol. Chem..

